# Salinomycin decreases feline sarcoma and carcinoma cell viability when combined with doxorubicin

**DOI:** 10.1186/s12917-019-1780-5

**Published:** 2019-01-24

**Authors:** Lucia Borlle, Abdo Dergham, Zacharie Wund, Brittany Zumbo, Teresa Southard, Kelly R. Hume

**Affiliations:** 1 Department of Clinical Sciences, Cornell University College of Veterinary Medicine, Ithaca, NY 14853 USA; 2 Department of Animal Sciences, Cornell University College of Agricultural and Life Sciences, Ithaca, NY 14853 USA; 3 Department of Biomedical Sciences, Cornell University College of Veterinary Medicine, Ithaca, NY 14853 USA

**Keywords:** Doxorubicin, Salinomycin, Feline, Chemoresistance, Solid tumors

## Abstract

**Background:**

Cancer is a significant health threat in cats. Chemoresistance is prevalent in solid tumors. The ionophore salinomycin has anti-cancer properties and may work synergistically with chemotherapeutics. The purpose of our study was to determine if salinomycin could decrease cancer cell viability when combined with doxorubicin in feline sarcoma and carcinoma cells.

**Results:**

We established two new feline injection-site sarcoma cell lines, B4 and C10, and confirmed their tumorigenic potential in athymic nude mice. B4 was more resistant to doxorubicin than C10. Dose-dependent effects were not observed until 92 μM in B4 cells (*p* = 0.0006) vs. 9.2 μM (*p* = 0.0004) in C10 cells. Dose-dependent effects of salinomycin were observed at 15 μM in B4 cells (*p* = 0.025) and at 10 μM in C10 cells (*p* = 0.020). Doxorubicin plus 5 μM salinomycin decreased viability of B4 cells compared to either agent alone, but only at supra-pharmacological doxorubicin concentrations. However, doxorubicin plus 5 μM salinomycin decreased viability of C10 cells compared to either agent alone at doxorubicin concentrations that can be achieved in vivo (1.84 and 4.6 μM, *p* < 0.004). In SCCF1 cells, dose-dependent effects of doxorubicin and salinomycin were observed at 9.2 (*p* = 0.036) and 2.5 (*p* = 0.0049) μM, respectively. When doxorubicin was combined with either 1, 2.5, or 5 μM of salinomycin in SCCF1 cells, dose-dependent effects of doxorubicin were observed at 9.2 (*p* = 0.0021), 4.6 (*p* = 0.0042), and 1.84 (p = 0.0021) μM, respectively. Combination index calculations for doxorubicin plus 2.5 and 5 μM salinomycin in SCCF1 cells were 0.4 and 0.6, respectively.

**Conclusions:**

We have developed two new feline sarcoma cell lines that can be used to study chemoresistance. We observed that salinomycin may potentiate (C10 cells) or work synergistically (SCCF1 cells) with doxorubicin in certain feline cancer cells. Further research is indicated to understand the mechanism of action of salinomycin in feline cancer cells as well as potential tolerability and toxicity in normal feline tissues.

**Electronic supplementary material:**

The online version of this article (10.1186/s12917-019-1780-5) contains supplementary material, which is available to authorized users.

## Background

Cancer is a leading cause of death in cats [1]. Associated clinical signs are often vague and the disease may be in advanced stages at the time of diagnosis, with limited options for loco-regional therapy. Tumor resistance to chemotherapy, both intrinsic and acquired, is a key cause of cancer-related mortality [2, 3]. It is often the only treatment option for patients with advanced cancer, and in veterinary medicine, it may also be the only option for families who cannot afford radiation therapy or radical surgeries, or for patients for whom radical surgery would result in significant morbidity and compromise function and quality of life. Tumors have genomic instability, and by the time they are clinically detectable have often developed mutations that contribute to chemoresistance in the absence of drug exposure [4, 5]. Chemoresistance varies between and within tumor types, with solid tumors such as injection site sarcomas (ISS) and oral squamous cell carcinoma (FOSCC) being particularly chemoresistant [6–13]. Targeting cellular mechanisms that signal for DNA damage recognition, cell cycle arrest, and DNA repair is one way to increase chemosensitivity [14]. Drugs that enhance cellular retention and/or promote nuclear transport or retention of cytotoxic drugs, inhibit cancer stem cells, or target the tumor microenvironment are other methods [3, 15–18].

Salinomycin is an ionophore antibiotic that has the capacity to increase the permeability of cytoplasmic and mitochondrial membranes through the efflux of potassium [19]. It is used in poultry as a coccidiostatic agent and in pigs and ruminants to increase nutrient absorption [20]. Gupta et al. first demonstrated the anti-tumor properties of salinomycin when screening thousands of drugs against breast cancer stem cells; salinomycin caused > 100 fold decrease in cell viability [21]. Research by separate groups found that cancer stem cells treated with salinomycin have Wnt/β-Catenin pathway inactivation [22] and that salinomycin can inhibit Wnt1-induced phosphorylation of LRP6 (lipoprotein receptor related protein 6) and downregulate expression of target genes like cyclin D1 [23]. Additional cellular alterations attributed to the anti-cancer activity of salinomycin include inhibition of the multidrug resistance transmembrane protein p-glycoprotein (PGP) [24–26] and G1-phase arrest with increased DNA damage, p21, and p53 [27]. Apoptosis occurred following salinomycin treatment in cells expressing high levels of Bcl-2 and PGP showing salinomycin may be able to work in chemoresistant cancers [28]. Liffers et al. investigated the in vitro combination of doxorubicin and salinomycin in fibrosarcoma, liposarcoma, and rhabdomyosarcoma cells [29]. Doxorubicin is a commonly used chemotherapeutic that affects a variety of changes in cells, including free radical production and inhibition of topoisomerase II [30]. Combination therapy with salinomycin was associated with increased *P53* expression and increased apoptotic activity [29].

The efficacy of salinomycin in feline cancer has not been investigated. Therefore, we developed ISS cell lines and tested whether salinomycin increased doxorubicin efficacy in these cells, as well as in FOSCC cells (SCCF1). Feline ISS is an aggressive tumor that arises at the site of injections with an unpredictable response to chemotherapy [31–33]. They are locally invasive and the first choice treatment is radical surgery [34, 35]. FOSCC is another cancer that is incurable in most cats and causes significant morbidity with clinical signs of severe pain and a functional obstruction to eating [36]. We investigated these tumor types in hopes of identifying a new strategy to increase chemosensitivity and improve outcomes for these cats.

## Results

### Immortalization and tumorigenicity of newly established feline ISS cell lines

**Fig. 1:**
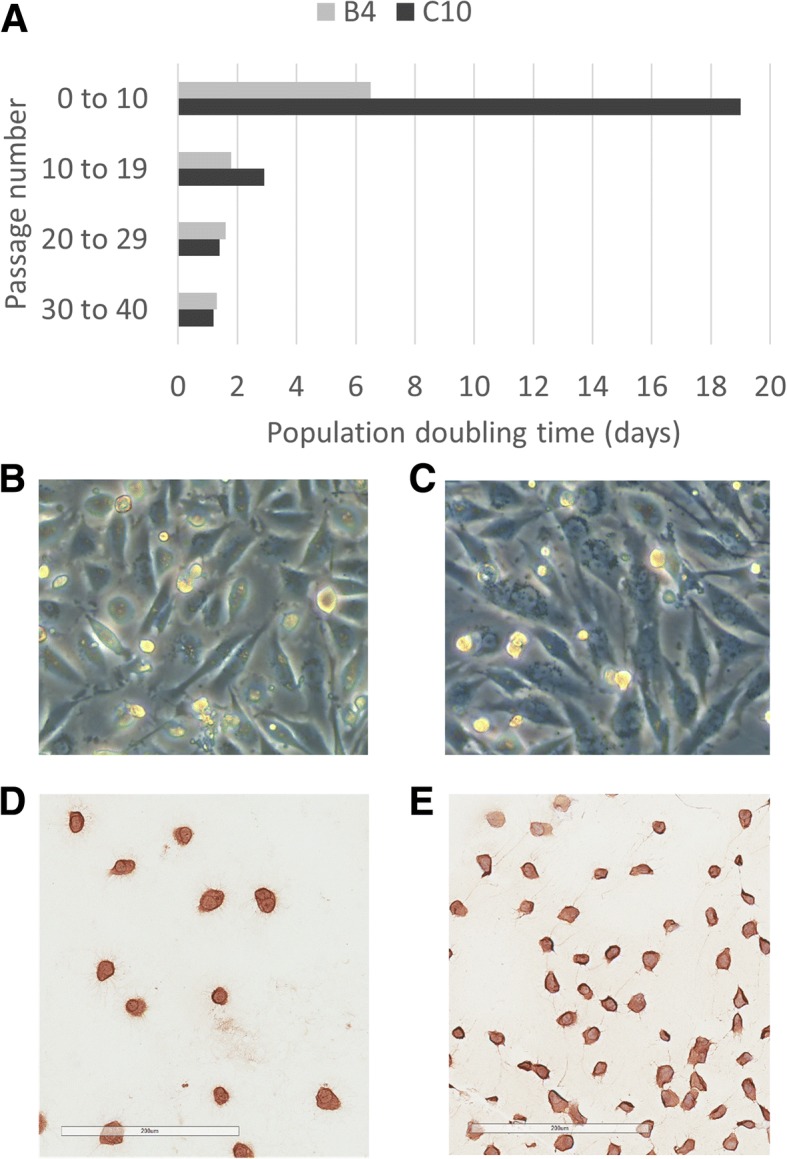
Features of B4 and C10 cells. **a**. B4 grew more quickly than C10 during early passages, with a population doubling time of 6.5 days compared to a population doubling time of 19 days. After passage 20, population doubling times between the two cell lines were similar. Both B4 (**b**) and C10 (**c**) cells display a spindled morphology in adherent, monolayer culture. Both B4 (**d**) and C10 (**e**) cells also display immunoreactivity for vimentin. Bar = 200 μm. No immunoreactivity was observed in the negative control

Cell lines B4 and C10 were established from two cats with ISS, diagnosed histologically as fibrosarcomas. Sample B4 was collected after euthanasia from a 13 year old male castrated cat with a recurrent injection site sarcoma on the right thorax. The tumor had been previously treated with palliative radiation therapy and various cytotoxic chemotherapeutics including doxorubicin. Sample C10 was collected from a 3 year old male cat at the time of incisional biopsy to confirm diagnosis. The tumor was located on the proximal right hindlimb; no prior anti-cancer therapy had been administered to this cat. Both B4 and C10 cell lines grew slowly initially, and then subsequently were observed to immortalize spontaneously. Both lines were grown continuously in culture until passage 40 (170 days in continuous culture for B4; 276 days in continuous culture for C10), at which time all remaining cells were frozen. Although the growth rates were initially quite different between the two cell lines, growth rates in later passages (i.e. between passage 20 and passage 40) were equivalent between the two cell lines with similar population doubling times (Fig. 1a). Cell line B4 reached 30 and 60 cumulative population doublings (PDs) after 106 and 145 days in culture, respectively. In contrast, cell line C10 did not reach 30 and 60 cumulative PDs until 191 and 233 days in culture, respectively. However, the time required to go from 30 to 60 population doublings was similar between cell lines (B4, 1.3 days; C10, 1.4 days). Spindle cell morphology was maintained throughout culture (Fig. 1b, c) and vimentin expression was confirmed in both cell lines (Fig. 1d, e).

**Fig. 2:**
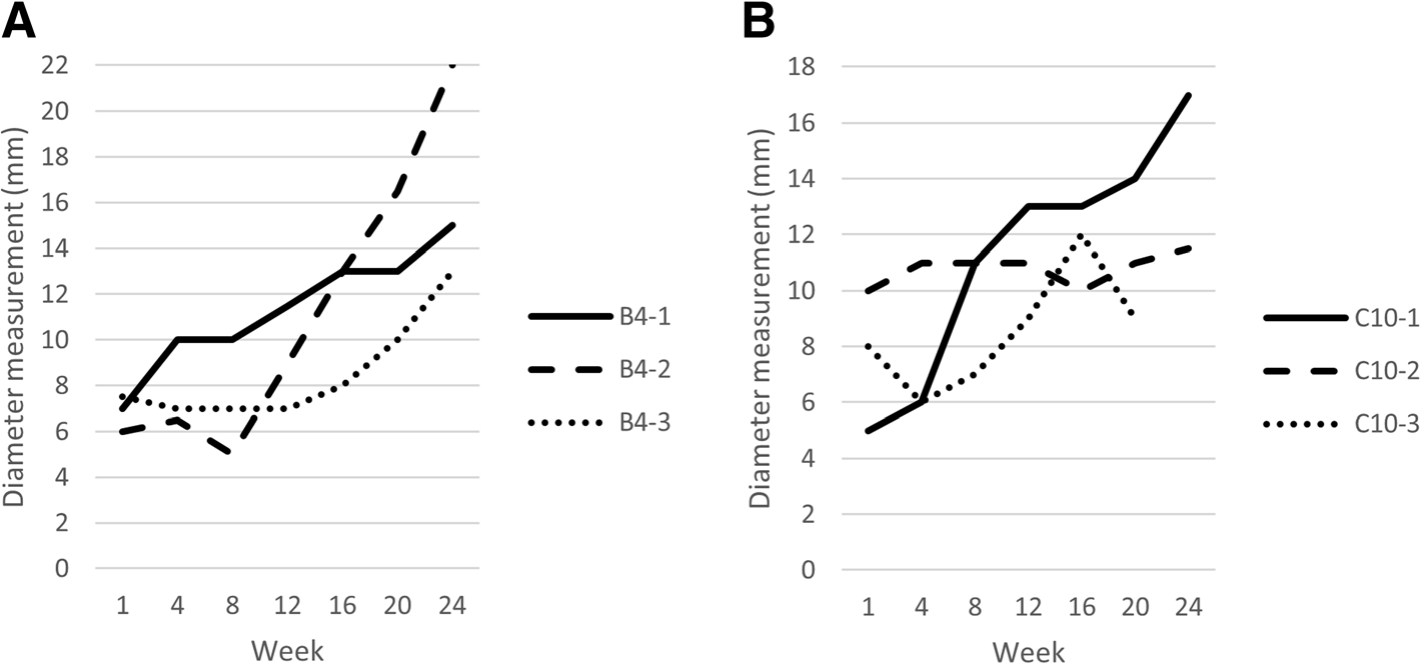
Diameter measurements of xenograft tumors over time. **a**. Measurements of tumors from 3 individual mice injected with B4 cells. **b**. Measurements of tumors from 3 individual mice injected with C10 cells. The tumor in mouse C10–3 began to regress after week 16 and was no longer palpable after week 20

**Fig. 3:**
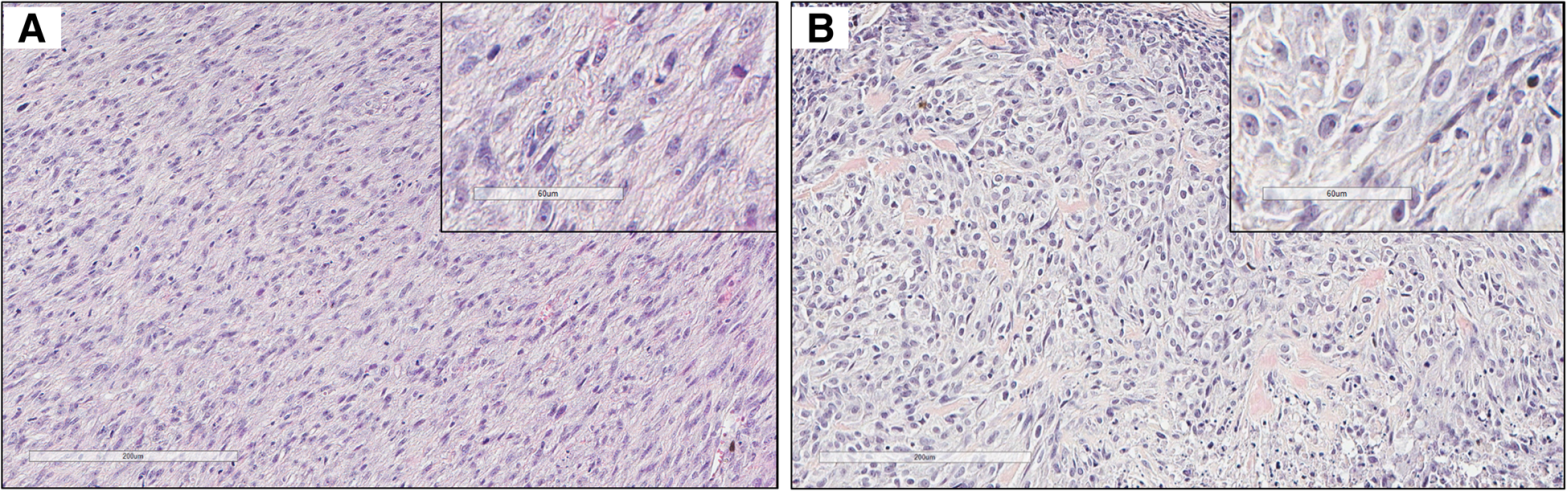
B4 and C10 ISS cells form sarcomas in a murine xenograft model. **a**. Sarcoma that formed at site of injection of 5 million B4 cells; this is tumor B4–2 (see Fig. 2). The tumor is composed of neoplastic spindle-shaped cells arranged in streams and bundles. The cells have moderate amounts of foamy to fibrillar cytoplasm and oval nuclei with finely stippled chromatin and 1–3 prominent nucleoli. Anisocytosis and anisokaryosis are moderate and there are 0–2 mitotic figures per 400x field. Hematoxylin and eosin staining; Bar = 200 µm. Inset bar = 60 µm. **b**. Sarcoma that formed at the site of injection of 5 million C10 cells; this is tumor C10–1 (see Fig. 2). The tumor is composed of polygonal to spindle-shaped neoplastic cells arranged in sheets and interlacing streams, interspersed with small aggregates of eosinophilic fibrillar material (collagen). The cells have moderate amounts of fibrillar cytoplasm and oval nuclei with finely-stippled chromatin and 1–2 prominent nucleoli. Anisocytosis and anisokaryosis are moderate and there is one mitotic figure per 400x field. Admixed with the neoplastic cells are peripheral infiltrates of lymphocytes. In less than 20% of the section, neoplastic cells have shrunken or fragmented nuclei (necrosis). Hematoxylin and eosin staining; Bar = 200 μm. Inset bar = 60 μm

**Fig. 4:**
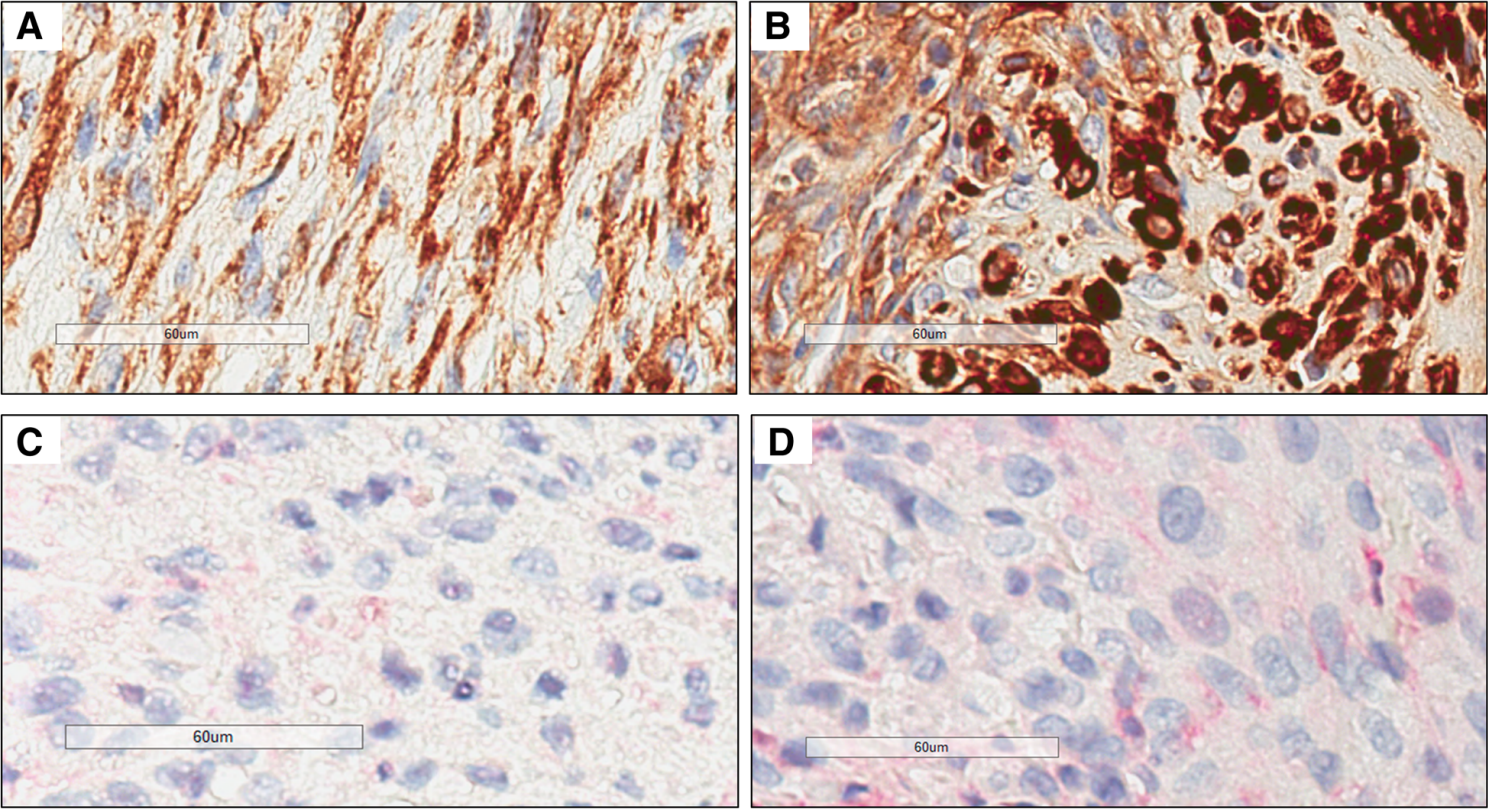
Murine xenograft sarcomas from ISS cells have vimentin immunoreactivity and lack cytokeratin immunoreactivity. Immunolabeling for vimentin (1:80, DAKO monoclonal mouse anti-vimentin, M7020) and cytokeratin (1:200, DAKO monoclonal mouse anti-human CKAE1/3 antibody, M3515) was performed by the Cornell University Animal Health Diagnostic Center Histology Laboratory (Ithaca, NY) using Leica Bond Max Automated IHC Staining System. Sarcomas that formed at the site of injection of either B4 (**a**) or C10 (**b**) cells showed moderate to strong cytoplasmic immunoreactivity for vimentin. No immunoreactivity was detected for cytokeratin in sarcomas that formed at the site of injection of either B4 (**c**) or C10 (**d**) cells. Bar = 60 μm

The tumorigenic potential of the cell lines was assessed in a xenograft model, with 5 million cells of each cell line injected subcutaneously into the right flank of athymic nude mice (*n* = 3 for each cell line). All injected mice developed subcutaneous tumors in the right flank that grew slowly over time (Fig. 2a-b). In one of the mice injected with C10 cells, the tumor regressed after week 16. No tumors developed in the left flank where control diluent was injected. The histologic appearance of the xenograft sarcomas generated from the two different cell lines was relatively similar (Fig. 3a-b; Additional file 1). Representative xenograft sarcoma samples from each cell line were evaluated for vimentin and cytokeratin expression. Both samples displayed immunoreactivity for vimentin (Fig. 4a-b); immunoreactivity for cytokeratin was not detected (Fig. 4c-d).

### Cell viability after doxorubicin, salinomycin and their combination

**Fig. 5:**
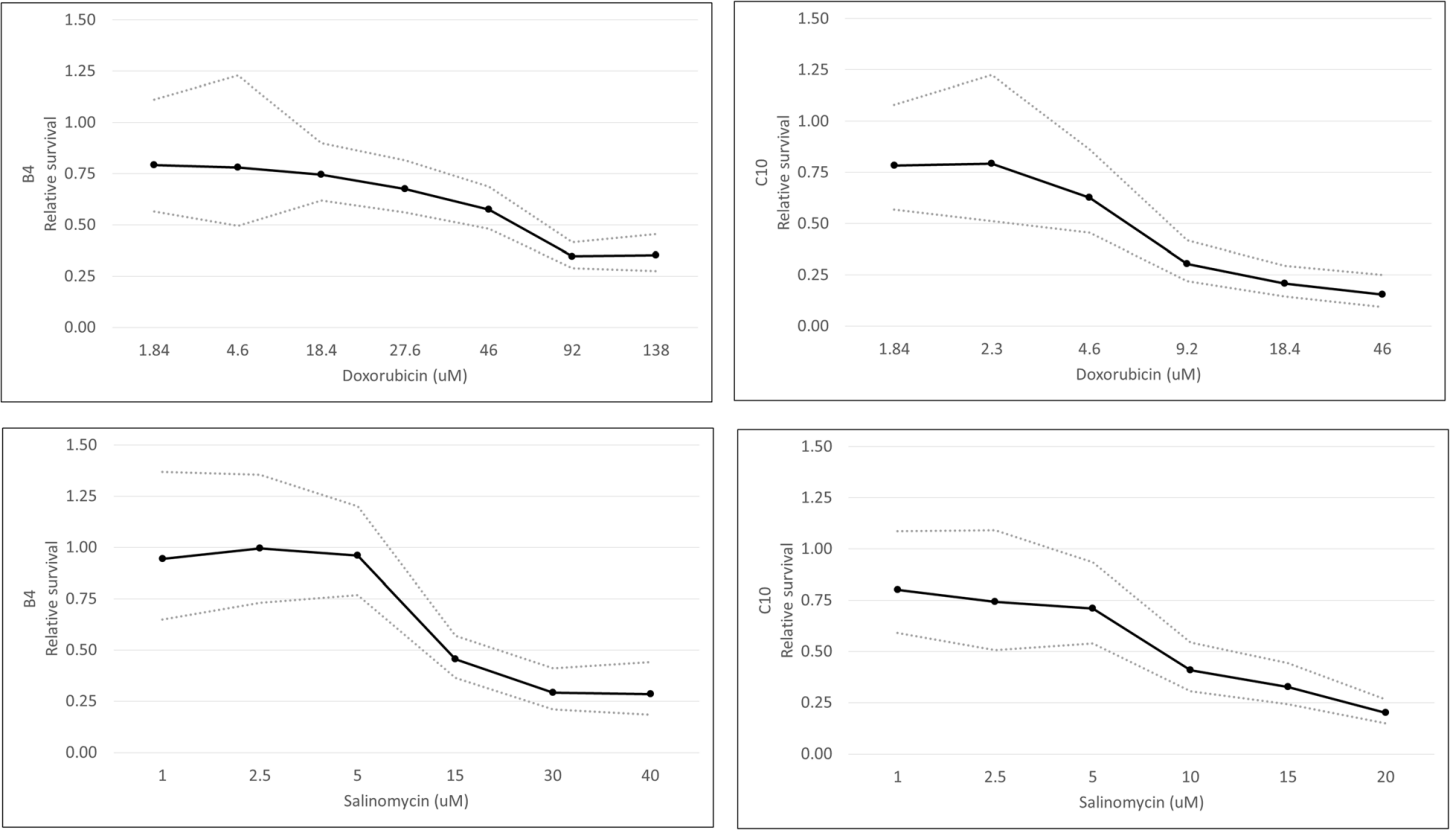
Dose-dependent effects of doxorubicin and salinomycin as single agents in B4 and C10 cells. MTT cell viability assays were performed in both B4 and C10 cells. Dose-dependent effects of doxorubicin were first observed in B4 cells at 92 μM and in C10 cells at 9.2 μM. Dose-dependent effects of salinomycin were first observed at 15 μM in B4 cells and at 10 μM in C10 cells. Solid dark lines depict the mean generated from statistical modeling for the indicated conditions. Dotted lines represent the upper and lower 95% confidence intervals. *P* values from individual comparisons are described in the text and are available in Additional file 2

To test whether salinomycin could enhance the effect of doxorubicin on ISS cells, MTT colorimetric assays were performed. Dose-dependent effects of doxorubicin and salinomycin as single agents were observed in both cell lines (Fig. 5). For B4 cells, cell viability following exposure to doxorubicin alone was evaluated in concentrations ranging from 0.184–138 μM. The IC50 based on these experiments was 47 μM (95% confidence interval, 20–110 μM). Dose-dependent effects of doxorubicin were first observed in B4 cells at 92 μM, which was significantly different from concentrations of 1.84–46 μM (*p* values ranging from < 0.0001 to 0.0288). For C10 cells, cell viability following exposure to doxorubicin alone was evaluated in concentrations ranging from 0.092–46 μM, and the IC50 was 7.4 μM (95% confidence interval, 6.0–9.2 μM). Dose-dependent effects of doxorubicin were first observed in C10 cells at 9.2 μM, which was significantly different from concentrations of 1.84–4.6 μM (*p* values ranging from 0.0004 to 0.016). Although the IC50 for doxorubicin alone is much lower in the C10 cells, results for both cell lines are above the reported C_max_ in cats, which ranged from 1.1–5.0 μM following a single clinically relevant dosage of either 25 mg/m^2^ or 1 mg/kg [37]. These results suggest doxorubicin may not have had significant clinical benefit as a single agent in the treatment of the tumors from which these cell lines were derived. The cat from which B4 was derived had received doxorubicin chemotherapy many months prior to sample collection and whether a clinical benefit was associated with this treatment is unknown (medical records not available for review). The cat from which C10 was derived did not receive doxorubicin as part of his clinical management.

Results following exposure to salinomycin were not as discrepant between the two cell lines (Fig. 5). Salinomycin concentrations ranging from 0.1–40 μM were evaluated in the B4 cells; the IC50 based on the results of these experiments was 11 μM (95% confidence interval, 8.4–13 μM). Dose-dependent effects of salinomycin were first observed in the B4 cells at 15 μM, which was significantly different from concentrations ranging from 1 to 5 μM (*p* values ranging from 0.0001 to 0.0247). Salinomycin concentrations ranging from 0.1–20 μM were evaluated in the C10 cells; the IC50 based on the results of these experiments was 9.9 μM (5.6–18 μM). Dose-dependent effects of salinomycin were first observed in the C10 cells at 10 μM, which was significantly different from 1 μM (*p* = 0.0204).

**Fig. 6:**
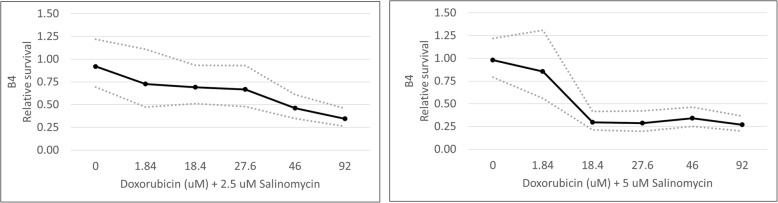
Dose-dependent effects of doxorubicin and salinomycin in combination in B4 cells. In MTT cell viability assays, dose-dependent effects of doxorubicin were first observed at 46 μM when administered in combination with 2.5 μM of salinomycin. Dose-dependent effects of doxorubicin were first observed at 18.4 μM when administered in combination with 5 μM of salinomycin. Solid dark lines depict the mean generated from statistical modeling for the indicated conditions. Dotted lines represent the upper and lower 95% confidence intervals. *P* values from individual comparisons are described in the text and are available in Additional file 2

To study whether salinomycin could potentiate the effects of doxorubicin, concentrations of 2.5 and 5 μM salinomycin were combined with a range of doxorubicin concentrations. These salinomycin concentrations were chosen because they had minimal impact on cell viability when used as single agents. For B4 cells, 2.5 and 5 μM salinomycin were combined with doxorubicin concentrations ranging from 1.84–92 μM (Fig. 6). The IC50 of doxorubicin when combined with 2.5 μM salinomycin in B4 cells was 36 μM (95% confidence interval, 26–48 μM). The IC50 of doxorubicin when combined with 5 μM of salinomycin in B4 cells was 6.0 μM (95% confidence interval, 3.9–9.1 μM). When combining doxorubicin with 2.5 μM salinomycin, combination treatment resulted in decreased B4 cell viability at doxorubicin concentrations as low as 46 μM compared to single agent 2.5 μM salinomycin (*p* < 0.0018). There was no difference compared to doxorubicin alone (*p* = 0.0672). Therefore, with combination treatment 46 μM doxorubicin potentiated the effect of 2.5 μM salinomycin, but not vice versa. Combination treatment with 5 μM salinomycin decreased B4 cell viability compared to either agent alone at doxorubicin concentrations as low as 18.4 μM (*p* < 0.018 compared to doxorubicin alone; *p* < 0.0018 compared to salinomycin alone). Although 5 μM salinomycin combination therapy resulted in decreased B4 cell viability compared with doxorubicin or salinomycin alone, the results were only observed at supra-pharmacological concentrations, therefore a combination index calculation was not performed.

**Fig. 7:**
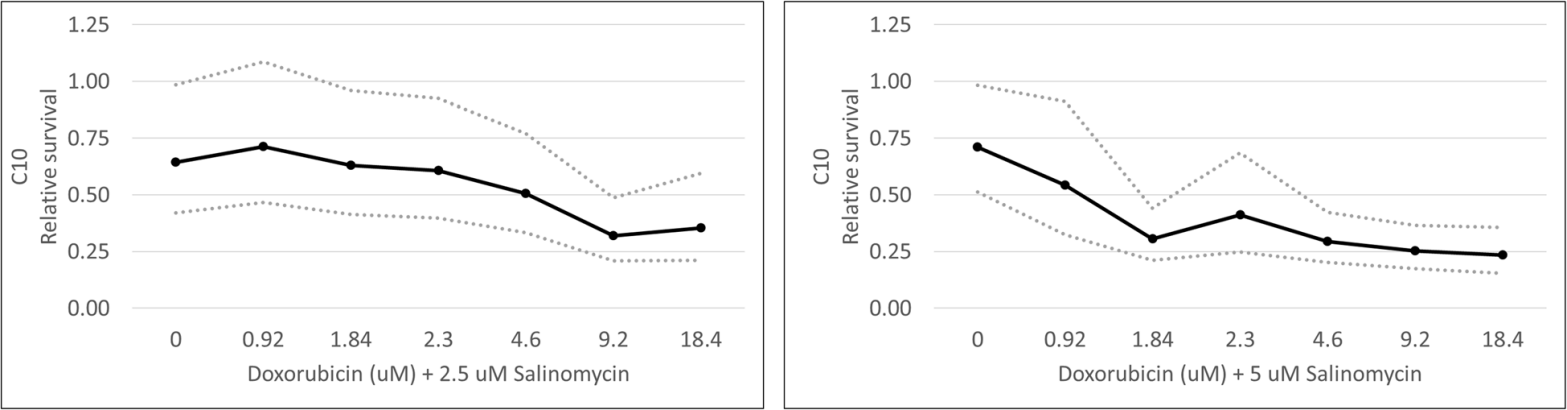
Dose-dependent effects of doxorubicin and salinomycin in combination in C10 cells. In MTT cell viability assays, no dose-dependent effects of doxorubicin were observed in combination with 2.5 μM of salinomycin. Dose-dependent effects of doxorubicin were observed at 1.84 and 4.6 μM or greater when administered in combination with 5 μM of salinomycin. Solid dark lines depict the mean generated from statistical modeling for the indicated conditions. Dotted lines represent the upper and lower 95% confidence intervals. *P* values from individual comparisons are described in the text and are available in Additional file 2

For C10 cells, 2.5 and 5 μM salinomycin were combined with doxorubicin concentrations ranging from 0.92–18.4 μM (Fig. 7). The IC50 of doxorubicin combined with 2.5 μM salinomycin in C10 cells was 2.5 μM (95% confidence interval, 1.6–3.9 μM). In order to calculate an IC50 for this combination that fit with the results we observed, data from doxorubicin at 4.6 μM had to be excluded. The IC50 of doxorubicin when combined with 5 μM salinomycin in C10 cells was 5.3 μM (95% confidence interval, 2.6–11 μM). Combination therapy with doxorubicin and 2.5 μM salinomycin did not decrease C10 cell viability in comparison to either agent alone (*p* > 0.05). However, combination therapy with 5 μM salinomycin did decrease C10 cell viability compared to either agent alone at doxorubicin concentrations of 1.84 and 4.6 μM (*p* < 0.004). Although combination therapy with 5 μM salinomycin resulted in decreased C10 cell viability at concentrations of doxorubicin that can be achieved in vivo, a combination index was not calculated due to overlapping IC50 confidence intervals between combination therapy and doxorubicin alone. (Although the IC50 confidence interval for 2.5 μM salinomycin combination therapy did not overlap with that of doxorubicin alone, no dose-dependent effects were observed, and thus a combination index was not calculated.)

**Fig. 8:**
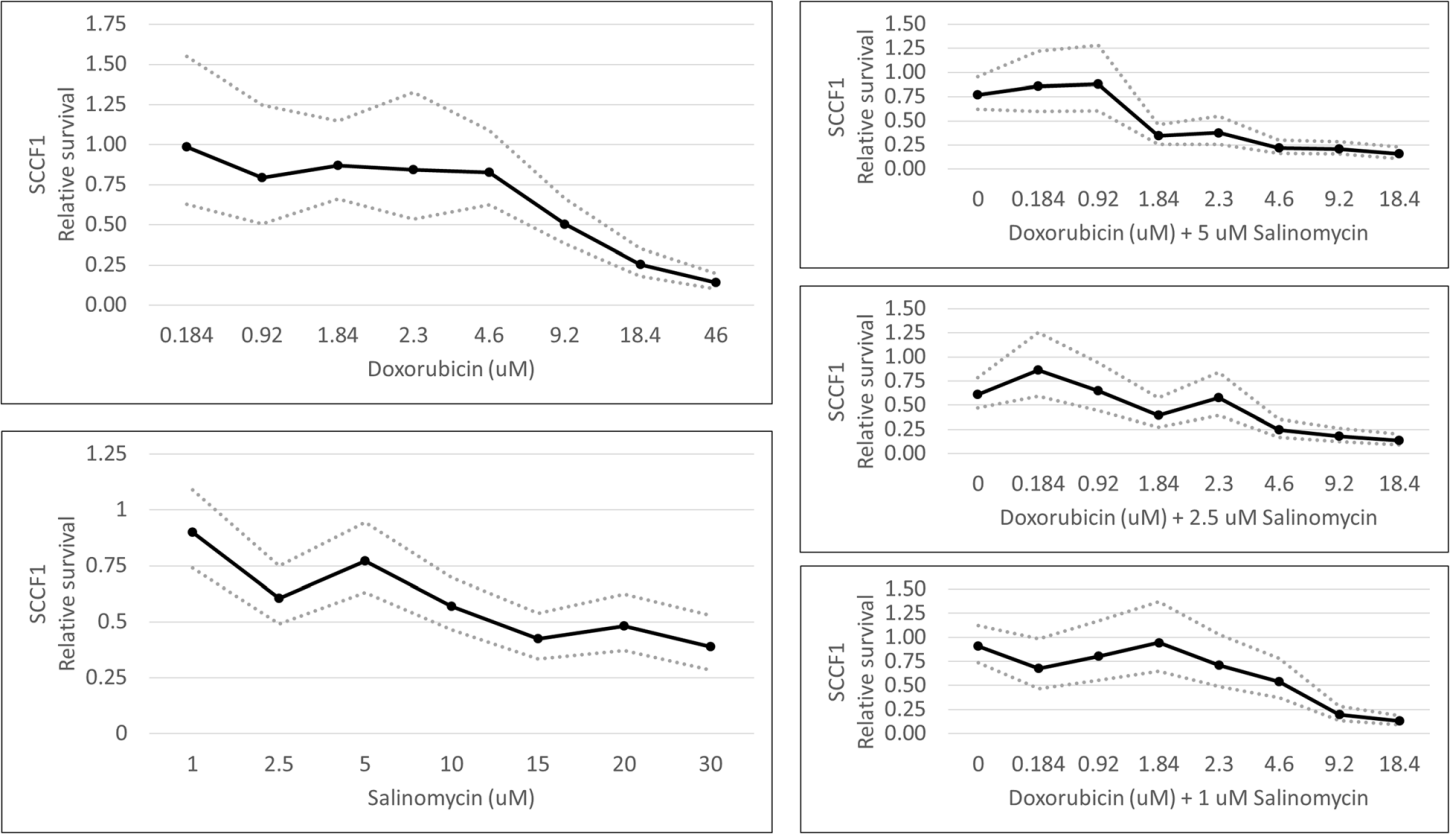
Single agent and combined effects of doxorubicin and salinomycin in SCCF1 cells. MTT cell viability assays were performed. Dose-dependent effects of doxorubicin were first observed at 9.2 μM. Dose-dependent effects of salinomycin were first observed at 2.5 μM. When combined with either 1, 2.5, or 5 μM of salinomycin, dose-dependent effects of doxorubicin were first observed at 9.2, 4.6, and 1.84 μM, respectively. Solid dark lines depict the mean generated from statistical modeling for the indicated conditions. Dotted lines represent the upper and lower 95% confidence intervals. *P* values from individual comparisons are described in the text and are available in Additional file 2

To test whether salinomycin could also potentiate the effects of doxorubicin in epithelial cancer cells, we evaluated the effects of salinomycin and doxorubicin on a feline oro-laryngeal squamous cell carcinoma cell line (SCCF1). As in the ISS cell lines, dose-dependent effects of doxorubicin and salinomycin as single agents were observed in the SCCF1 cells (Fig. 8). Doxorubicin concentrations ranging from 0.184–46 μM were tested; the IC50 calculated from these results was 8.9 μM (95% confidence interval, 7.2–11.0 μM). Dose-dependent effects of doxorubicin were first observed at 9.2 μM, which was significantly different from 1.84 μM (*p* = 0.036). Salinomycin concentrations ranging from 1–30 μM were tested; the IC50 calculated from these results was 11 μM (95% confidence interval, 7.5–16 μM). In order to calculate an IC50 that fit with the results we observed, data from salinomycin at 1 μM had to be excluded. Dose-dependent effects of salinomycin were first observed at 2.5 μM, which was significantly different from 1 μM (*p* = 0.0049).

To study the effect of combination therapy in SCCF1 cells, concentrations of 2.5 and 5 μM salinomycin were combined with doxorubicin concentrations ranging from 0.184–18.4 μM (Fig. 8). The IC50 calculated from results of the 2.5 μM combination experiments was 1.7 μM (95% confidence interval, 1.0–3.0 µM). The IC50 calculated from results of the 5 μM combination experiments was 1.6 μM (95% confidence interval, 1.3–1.9 μM). When combining doxorubicin with 2.5 μM of salinomycin, SCCF1 cell viability decreased compared to either agent alone at doxorubicin concentrations of 4.6 (*p* < 0.005) and 9.2 μM (*p* < 0.003). The combination index for doxorubicin plus 2.5 μM salinomycin in SCCF1 cells is 0.4. When combining doxorubicin with 5 μM of salinomycin, SCCF1 cell viability decreased compared to either agent alone at doxorubicin concentrations ranging from 1.84–9.2 μM (*p* values ranging from < 0.0021 to 0.042). The combination index for doxorubicin plus 5 μM salinomycin is 0.6.

Because dose-dependent effects were observed with 2.5 μM salinomycin combination therapy in SCCF1 cells, combination therapy with 1 μM salinomycin was also evaluated (Fig. 8). Doxorubicin concentrations again ranged from 0.184–18.4 μM; the IC50 calculated from these results was 5.2 μM (95% confidence interval, 4.0–6.7 μM). With 1 μM salinomycin combination therapy, SCCF1 cell viability decreased compared to either agent alone at a doxorubicin concentration of 9.2 μM (*p* = 0.0042 in comparison to doxorubicin alone; *p* = 0.0021 in comparison to salinomycin alone). A combination index calculation for this combination was not performed, because the effect was only observed at a supra-pharmacological doxorubicin concentration.

Additional file 2 contains the viability results and *p* values for all drug concentration comparisons that were found to be significant in our statistical modeling.

## Discussion

In this study, we established ISS cell lines in order to study chemoresistance in feline cancer. We found that both B4 and C10 were resistant to single agent doxorubicin, given that dose-dependent effects were not observed with concentrations that can be achieved in vivo. C10 was derived from a naïve tumor that had not previously been treated with any anti-cancer therapeutics and B4 was generated from a tumor that had been exposed to many different chemotherapeutic agents, including doxorubicin. The prior exposure of B4 cells to multiple chemotherapeutics should be taken into consideration when using this cell line to investigate efficacy of novel therapeutics. Sarcomas formed at the sites of injection of both B4 and C10 ISS cells in athymic nude mice, confirming the tumorigenic potential of both cell lines (Figs. 2, 3, 4; Additional file 1).

Similar to data with cytotoxic chemotherapeutics in human cancer cells [22, 24, 29, 38–40], we found that salinomycin can inhibit feline cancer cell viability. The observed effects of salinomycin were relatively similar across all 3 cell lines, with minimal loss of cell viability at concentrations ≤5 μM, and IC50s ranging from 9.9 to 11 μM. Dose-dependent potentiation of single agent effects was observed when salinomycin was combined with doxorubicin in all 3 cell lines tested. However, in the B4 cell line most resistant to single agent doxorubicin (i.e. highest IC50), potentiation was only observed at supra-pharmacological doxorubicin concentrations. This finding suggests combination therapy with salinomycin may not be useful for tumors previously exposed to doxorubicin. Potentiation was observed in C10 cells when doxorubicin concentrations of 1.84 and 4.6 μM or greater were combined with 5 μM salinomycin. Because doxorubicin concentrations of 5 μM or less can be achieved in vivo, this combination may have clinical relevance, provided 5 μM of salinomycin can be achieved in vivo. The SCCF1 cells were the most sensitive to combination therapy, with potential synergism observed when doxorubicin was combined with salinomycin concentrations of either 2.5 or 5 μM. Based on our results, salinomycin combination therapy may be more beneficial in feline epithelial cancers, as compared to mesenchymal cancers. However, too few cell lines were evaluated for any definitive conclusions.

This work is, to our knowledge, the first testing of salinomycin in feline cancer cells. Salinomycin is an FDA-approved medicated feed additive for use in poultry. Repurposing the drug for use in companion animals would likely be faster and less costly than that associated with a novel drug, bringing more affordable and immediate changes for affected patients and their families [41]. There is considerable variability in tolerability of salinomycin across mammals, with significant adverse events associated with accidental toxic overdoses [20, 42]. One-percent of cats unintentionally exposed to contaminated dry cat food (16–21 mg/kg contaminant) developed a polyneuropathy that resulted in death of some cats (percentage unknown) whereas clinical signs resolved in others; provocation testing did not result in clinical signs in two cats tested, suggesting adverse events may not result in greater morbidity or mortality than that seen with cytotoxic chemotherapeutics [42]. No mortality was observed in C57Bl/6 mice that received 5 mg/kg daily for 30 days. C_max_ was 1.72 μM and the drug was almost completely eliminated within 5 h [43]. Although the mice did not develop paresis at this dosage, they did develop a sensory neuropathy that was ameliorated with concurrent pharmacological inhibition of mitochondrial Na^+^/Ca^2+^-exchangers. Interestingly, salinomycin plasma concentrations as high as 100 μM have been reported in poultry [44]. Given the results of our in vitro evaluation, salinomycin concentrations of 2.5 μM or greater may be necessary to observe clinically beneficial effects in feline cancer. The authors are not aware of any salinomycin pharmacokinetic studies that have been done in cats. Successful clinical implementation of salinomycin in the treatment of companion animal cancer will require additional research to understand mechanism and applicability to multiple tumor types, as well as efforts to predict tolerability and optimize dosage schemes, before clinical testing can be instituted. In vitro analysis of the effects of combination therapy on non-neoplastic cells is also indicated.

The ISS cell lines (B4 and C10) we developed are suitable for in vivo testing in a xenograft model. A variety of mouse strains can be used in xenograft testing. Although our cells formed sarcomas in CD-1 nude mice, one tumor did regress and growth was relatively slow overall (Fig. 2). This strain of mice lacks thymic tissue but has mature B cells and NK cells, which likely contributed to the tumor regression and slow growth rates we observed. Models with higher immunodeficiency such as NOD-SCID, NSG, or NOG may be more useful in future research [45–47]. Although our in vitro data suggests that these cell lines are relatively resistant to single agent doxorubicin chemotherapy, this should be confirmed through in vivo evaluation in a similar xenograft model.

## Conclusions

In our work we established 2 new feline ISS cell lines, B4 and C10, from primary tumors. We demonstrated the xenograft potential of these lines, which can now be used to identify new therapeutic strategies. In this vein, we investigated the potential for the ionophore salinomycin to increase chemosensitivity to doxorubicin and identified potentiation of single agent effects at doxorubicin concentrations achievable in vivo in the C10, but not the B4 cells. Possible synergy was observed in the FOSCC line, SCCF1. Our results provide the foundation for future studies aimed at repurposing salinomycin as an anti-cancer agent in companion animals.

## Methods

Animal research was performed according to a protocol approved by the Cornell University Institutional Animal Care and Use Committee (# 2012–0112). Informed consent was obtained when applicable. Best practices of veterinary care were followed. Applicable animal care occurred in accordance with Guide for the Care and Use of Laboratory Animals [48].

### Cell lines

ISS cell lines B4 and C10 were established in our laboratory from incisional biopsy specimens collected from privately owned cats. Concurrently collected adjacent tissue biopsy specimens from the same cats were also evaluated by a board-certified veterinary anatomic pathologist (TS) and diagnosed as fibrosarcomas. (These two histological samples were included in a separate study on the presence of DNA damage in ISS [49]). To establish the cell lines, tissue specimens were washed twice in PBS (phosphate buffered saline), once in trypsin, and then aseptically minced into multiple 2 mm tissue pieces that were plated in individual wells on a 12 well plate in DMEM media supplemented with 15–20% FBS (Sigma-Aldrich), 1% L-Glutamine 200 mM (Corning), 1% non-essential amino acids (Gibco), and 1% antibiotic-antimycotic (amphotericin B, penicillin, streptomycin; Gibco). Explants were removed when adherent monolayers of spindled cells were apparent. Adherent cells were passaged when confluent to sequentially larger tissue culture dishes. Once cells were maintained in 10 cm diameter dishes, they were counted each time they were passaged and population doublings were calculated. Adherent cells were harvested from tissue culture plates via trypsinization and counted using a standard hemacytometer (Bright-Line, Hausser Scientific). A proportion of passaged cells were periodically frozen in FBS with DMSO (10%) in liquid nitrogen or at − 80 °C to allow “stock” for use in future experiments. Immunolabeling for vimentin was performed as described elsewhere [50].

SCCF1 was made available as a gift to Cornell University from Dr. Thomas Rosol, The Ohio State University College of Veterinary Medicine, Columbus, OH 43210 [51].

All 3 cells lines (B4, C10, and SCCF1) were determined to be feline in origin and free of contamination of DNA from human, rat, mouse, hamster, and dog. This was performed independently by the Flint Animal Cancer Center Colorado State University Cell Validation Core (PI: Duval) based on methods previously reported [52, 53]. The certification of analysis is available from the corresponding author upon request. Cell lines were also determined to be free of *Mycoplasma* contamination using the Venor™ GeM Mycoplasma Detection Kit (Sigma, MP0025) according to manufacturer’s instructions.

### Xenografts

Heterozygous female CD-1 nude mice (Charles River, strain code 087) were intercrossed to homozygous male CD-1 nude mice (Charles River, strain code 086) to generate homozygous, athymic, nude males for use in xenograft experiments. The potential for B4 (passage 42) and C10 (passage 38) cells to form xenograft tumors was tested. Each cell line was tested in three different mice; mice that received B4 cells were littermates 47 days in age, and mice that received C10 were littermates 54 days in age. Mice were anesthetized with inhalant isoflurane for the injections. Each mouse received 5 million cells subcutaneously in the right flank. Cells were diluted in 200 μL of a 1:1 ratio of PBS and matrigel basement membrane mix (Corning). For control, 200 μl of 1:1 ratio of PBS and matrigel without cells was injected subcutaneously in the left flank. Tumor development was monitored for 6 months and diameter measurements were obtained weekly with manual calipers. Mice were euthanized via C0_2_ asphyxiation (per IACUC approved protocol), necropsies were performed, and subcutaneous tumors were harvested and fixed in 4% paraformaldehyde. Paraffin-embedded sections were stained with hematoxylin and eosin and analyzed by a board-certified veterinary pathologist (TS).

### Chemotherapeutics

Stock solutions were prepared, sterile-filtered, and kept frozen at − 20 °C until needed. Doxorubicin (Sigma-Aldrich) was prepared in saline (2 mg/ml) and salinomycin (Sigma-Aldrich) was prepared in DMSO (5 mM). Aliquots of stock solution were diluted as needed in saline (for doxorubicin) or DMEM (for salinomycin) for use in experiments.

### Cell viability assays

Viability experiments were performed using cells between passages 20 and 40, which is when both cell lines were observed to have similar population doubling times. To determine the effects of doxorubicin and salinomycin on cell viability, colorimetric MTT (3-(4,5-dimethylthiazol-2-yl)-2,5-diphenyltetrazolium bromide) assays were used. ISS cells B4 and C10 were plated at a density of 30,000 cells per well in a 96 well plate. SCCF1 cells were plated at a density of 15,000. These densities were chosen to give similar control values in both cell lines. Cells were allowed to adhere and proliferate for 48 h. They were then washed in PBS and treated with concentrations as indicated of doxorubicin, salinomycin, or both. Control cells for each experimental condition were treated with the same concentration of the relevant diluent(s). Cells were exposed to media with drug or control for 48 h. Each experiment involved triplicates of evaluated conditions. Evaluated conditions were tested in at least 2 independent experiments. MTT (5 μg/ml) was freshly prepared in PBS for each experiment. Cells were exposed to MTT for 2 h and then lysed in isopropanol for 1 h. Spectrophotometric readings at 570 and 690 nm were obtained on a SpectraMax 190 Microplate Reader (Molecular Devices). For each well, background absorbance at 690 nm was subtracted from absorbance at 570 nm. Means for each control condition were generated. Absorbance from individual treatment wells were reported relative to the appropriate mean control condition.

### IC50 and combination index (CI) calculations

In order to evaluate for synergy associated with combination therapy, IC50 values and their associated 95% confidence intervals were determined for each cell line for salinomycin alone, doxorubicin alone, and doxorubicin when combined with a specific concentration of salinomycin. This was done using nonlinear modeling and four parameter logistic fit (JMP, Version <JMP Pro 13.1.0>; SAS Institute Inc., 2016). The IC50 value represents an estimate of the drug concentration at which 50% of viability is inhibited. When results of our statistical modeling (described below) revealed significant differences (*p* < 0.05) between combination therapy and doxorubicin alone at concentrations achievable in vivo, the IC50 values were used in combination index (CI) calculations according to the formula: a/A + b/B = CI, where a = IC50 of the combination with salinomycin at “b”, A = IC50 of doxorubicin alone, b = the concentration of salinomycin used in “a”, and B = IC50 of salinomycin alone [54, 55]. Drugs are antagonistic when the result is greater than 1, additive if equal to 1, and synergistic when less than 1. This method was chosen because our goal was to analyze the combination effects of salinomycin at concentrations that had minimal impact on cell viability.

### Statistical analysis

For each cell line, viability was determined following exposure to multiple concentrations of doxorubicin and salinomycin, both alone and in combination. Within an individual experiment each concentration was tested in triplicate, and each concentration was evaluated in at least two independent experiments. Due to the multitude of concentrations tested, it was not possible to test all concentrations/combinations in every experiment. For each cell line we first determined whether dose-dependent effects were present with either doxorubicin alone, salinomycin alone, or with doxorubicin and salinomycin in combination. Because replicates of multiple concentrations were evaluated within an experiment, and multiple experiments were performed, multilevel statistical modeling was performed for each cell line and each treatment condition. To account for the testing of different concentrations in different experiments, experiment date and concentration group within the experiment date (3 replicates per group) were considered as random effects within the model. Fixed effects within the model were either doxorubicin concentration (for evaluating doxorubicin alone), salinomycin concentration (for evaluating salinomycin alone), or the combination of both doxorubicin and salinomycin concentrations (for evaluating the effect of combination therapy). The response variable of relative survival was log transformed to meet model assumptions. In order to determine which specific drug concentrations were associated with differences in viability, post hoc evaluation of multiple comparisons was performed using either Tukey’s test (single agent therapy) or Bonferroni correction (combination therapy). *P* < 0.05 was considered significant. All modeling was performed using statistical software (JMP, Version <JMP Pro 13.1.0>; SAS Institute Inc., 2016). Back calculations of the transformed response variable and associated confidence intervals were performed in Microsoft Excel (2013).

## Additional files

Additional file 1: contains H&E images of sarcomas B4–1, B4–3, and C10–2. (PPTX 8662 kb)

Additional file 2: contains the results of our statistical modeling, with tables of *p* values when significant differences in viability were identified between treatment conditions. (XLSX 24 kb)

## Abbreviations

CI: Combination index
FOSCC: Feline oral squamous cell carcinoma
ISS: Injection site sarcoma
MTT: 3-(4,5-dimethylthiazol-2-yl)-2,5-diphenyltetrazolium bromide
PBS: Phosphate buffered saline
