# Supplementary figures and images for: Salinomycin decreases feline sarcoma and carcinoma cell viability when combined with doxorubicin
Source: BMC Vet Res. 2019 Jan 24;15:36. doi: 10.1186/s12917-019-1780-5 (PMC6346515; doi:10.1186/s12917-019-1780-5)

## Slide 1
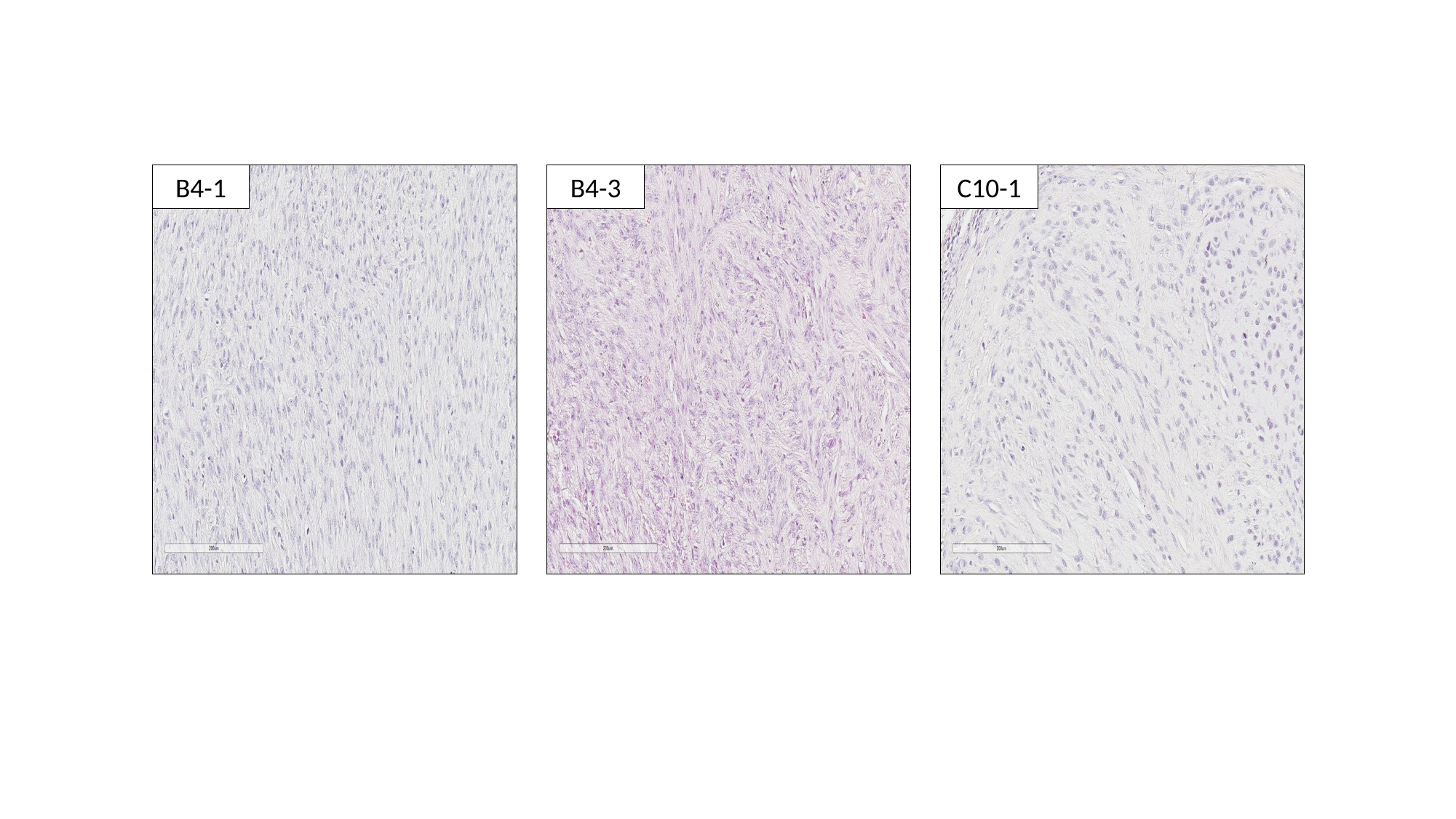

B4-1
B4-3
C10-1

Supplement: Supplementary file 1 — contains H&E images of sarcomas B4–1, B4–3, and C10–2. (PPTX 8662 kb) [file 12917_2019_1780_MOESM1_ESM.pptx]
